# Pattern recognition with machine learning on optical microscopy images of typical metallurgical microstructures

**DOI:** 10.1038/s41598-018-20438-6

**Published:** 2018-02-01

**Authors:** Dmitry S. Bulgarevich, Susumu Tsukamoto, Tadashi Kasuya, Masahiko Demura, Makoto Watanabe

**Affiliations:** 1 Research and Services Division of Materials Data and Integrated System, National Institute for Materials Science, 1-2-1 Sengen, Tsukuba, Ibaraki 305-0047 Japan; 2 Research Center for Development of Far-Infrared Region, University of Fukui, Fukui, 3-9-1, Bunkyo, 910-8507 Japan; 3 School of Engineering, The University of Tokyo, 7-3-1 Hongo, Bunkyo-ku, Tokyo 113-8656 Japan

## Abstract

For advanced materials characterization, a novel and extremely effective approach of pattern recognition in optical microscopic images of steels is demonstrated. It is based on fast Random Forest statistical algorithm of machine learning for reliable and automated segmentation of typical steel microstructures. Their percentage and location areas excellently agreed between machine learning and manual examination results. The accurate microstructure pattern recognition/segmentation technique in combination with other suitable mathematical methods of image processing and analysis can help to handle the large volumes of image data in a short time for quality control and for the quest of new steels with desirable properties.

## Introduction

**Figure 1:**
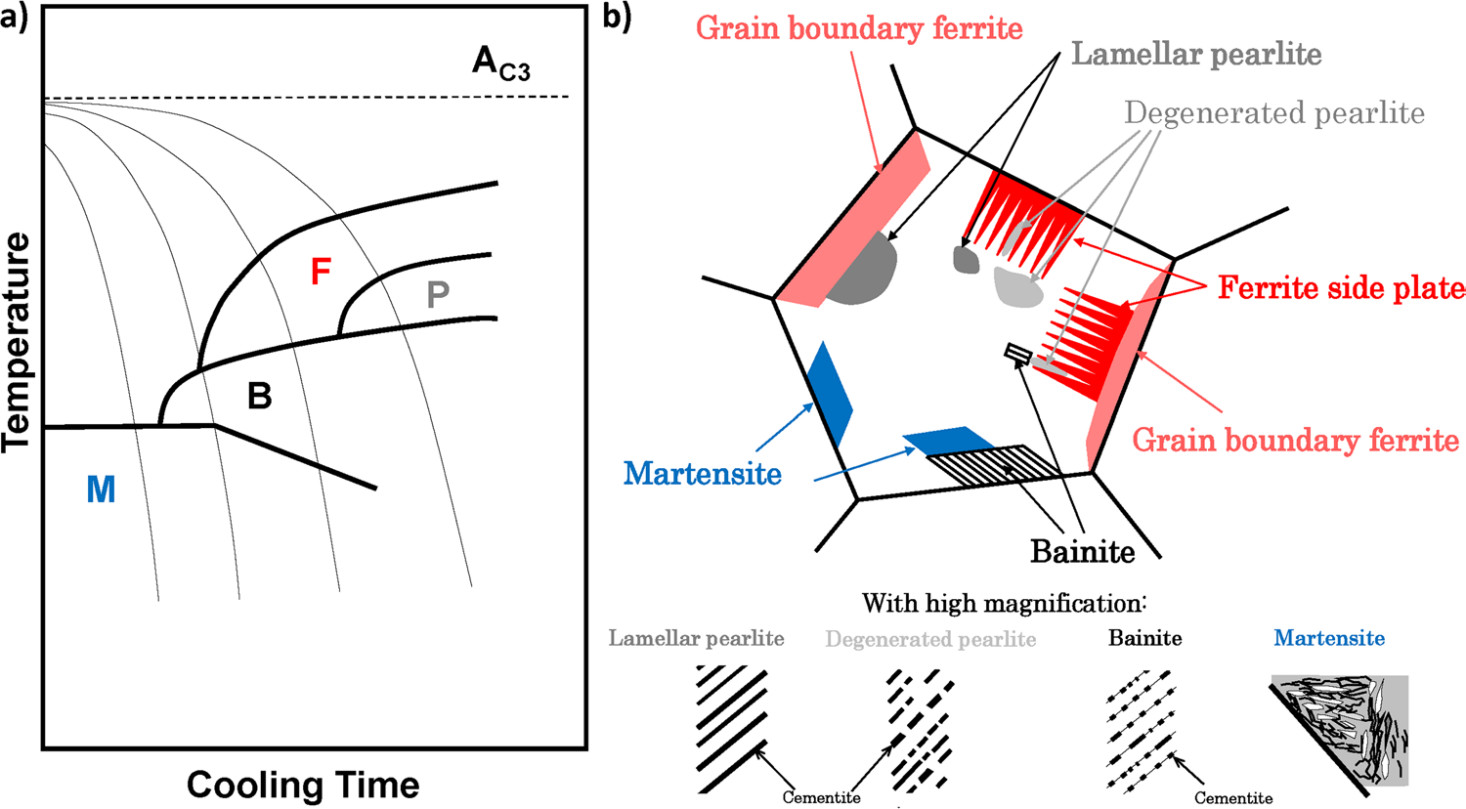
(**a**) The typical CCT diagrams with depicted regions of formed microstructures in low-carbon A-type structural steels. (**b**) The schematic drawing of different steel microstructures, which are formed in synthetic weld-heat affected zone during CCT process. See text for more details and abbreviations.

Depending on cooling rate of steels, the ferrite (F), pearlite (P), bainite (B), and martensite (M) microstructures could be formed due to the displacive and reconstructive transformations of austenite (A) crystal structure, which are accompanied with cementite precipitation at different diffusion rates (except for M)^1^. This is shown in Fig. 1(a) with continuous cooling transformation (CCT) diagrams^2^. For example, the various combinations of such microstructures could be formed and observed at different microscope magnifications in a weld heat-affected zone (HAZ). The appearance and additional nomenclature for some of them related to present work are shown in Fig. 1(b), which are not limited to welding process only. Here it must be stressed that Fig. 1(b) is for summary only since not all different microstructures could be observed at once in particular steel as can be seen from Fig. 1(a).

It is well known that the volume fraction, the dimension, and the morphology of these microstructure phases are greatly responsible for the mechanical properties of metallurgical materials^3–5^. For instance, the sensitivity to weld-cold cracking is in order of M > B > F volume fractions. So far, however, such microstructural information was quantified manually for industrial or scientific purposes. Obviously, this was/is a very time-consuming process even for skilful personal. In addition, it must be noted that with the manual quantification, it is virtually impossible to obtain the statistical information on phases having the complex spatial distributions.

**Figure 2:**
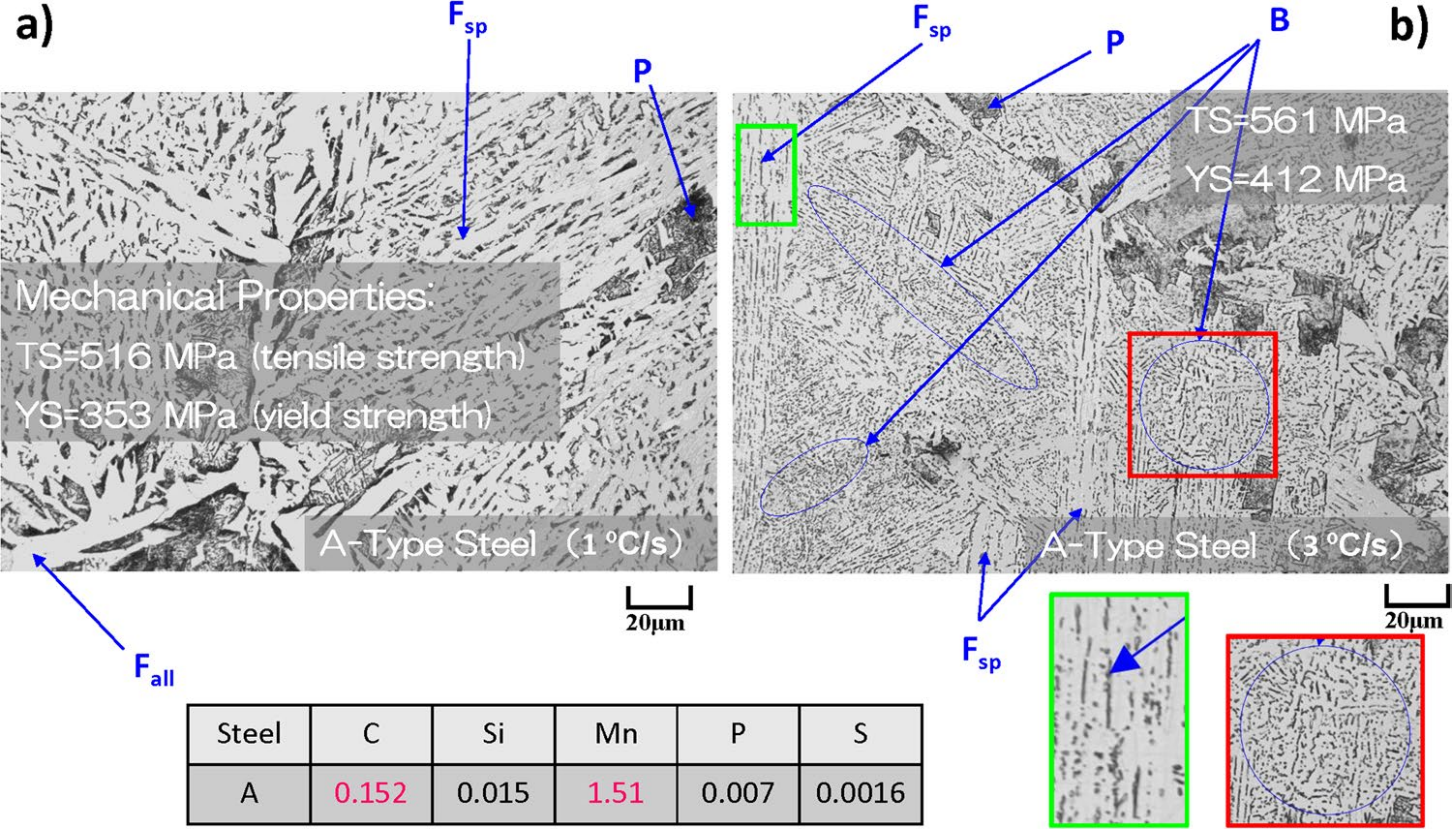
The examples of complex microstructure patterns observed in optical microscopy images of two A-steels with same composition, but obtained with different cooling rates of 1 °C/s (**a**) and 3 °C/s (**b**) from 1400 °C and having different mechanical properties. The manual identification of different microstructures is shown with arrows.

The outline of complexity of the problem is depicted in Fig. 2(a,b), where the optical microscopy images of steel microstructures with same chemical composition, but cooled at 1 and 3 °C/s are shown. In Fig. 2(a), the P, ferrite side plate (F_sp_), and grain boundary allotriomorphic ferrite (F_all_) could be detected compared to additional B phase in Fig. 2(b). These differences are manifested in changes of their macroscopic mechanical properties, i.e. the higher tensile and yield strengths for steel with B. In Fig. 2 and subsequent discussion, the fine colony P and M-A constituent are included into the F_sp_ since they could nucleate together in some cases between F_sp_. In present samples, it is difficult to distinguish them by optical microscopy alone. In addition, the lamellar and degenerated P types from Fig. 1(b) are assigned to P. The lower B also cannot be seen with present optical magnification without scanning electron microscopy (SEM), so B corresponds to upper B in present work. If necessary to get more detailed information, the method of image analysis presented below can be equally applied to SEM images.

Typically, the volume fractions of the phases are estimated with linear analysis. In this method, the length of all intercepts for a certain phase is measured along the straight line drawn on sample image. If the line length is long enough, it is assumed that ratio of the summed length for particular phase to the total line length is equal to the volume fraction of this phase. In metallurgy field, there is a great demand to automate the image processing on huge data volumes, which are already available and continue to grow. In principle, on modern PC workstations and supercomputers, the sophisticated machine learning techniques^6^ for pattern recognitions in combination with other suitable mathematical methods of image processing and analysis^7–9^ can help to handle such volumes of image data in a short time for materials quality control, for establishing of microstructure/mechanical property relations, and for the quest on new materials with desirable characteristics, i.e. for the goals of materials informatics. For example, there has been a dramatic progress in automated analysis of medical images with different segmentation techniques^10–12^.

However, it must be stressed that for microscopy images of metallurgical samples with complex microstructures (as in Fig. 2) we are not aware of reported image segmentations with accurate automatic techniques. In reported attempts, the different segmentation methods, algorithms, or microstructure descriptors were used or derived, which were limited to particular problem, were not at the pixel level scale, or classification qualities were not examined in details^13–21^. Here it should be stressed that for industrial applications the automated phase segmentation quality must be in pair or better with manual analysis by experts. In this respect, the reported image segmentations by using the multilayer perceptron in backpropagation artificial neural network stands out, though the segmented microstructure types and their complexities were less demanding compared to present work^22–26^. The direct comparison of several machine learning classification techniques on image segmentation of graphite particles in metallurgical materials were also studied, but didn’t include the methods used here^27^.

Accordingly, we are presenting the application of several image segmentation methods, which are suitable for discussed challenges and requirements. The segmentation with machine learning could be a core in many practical protocols for analysis of metallurgical samples due to its versatility and accuracy, but not the single one as it will be demonstrated below.

## Principle

In this regard, we had implemented the particular machine learning with Random Forest statistical algorithm^28–30^ to the typical image analysis demands in metallurgy field. In fact, there are several reports of successful use of this algorithm in other fields such as biological and medical sciences^31–33^. In short, this method creates an appropriate sequences of applied image filters which can be used to assign each pixel in sample image to the particular class (j = A, B, C, …, n) with highest probability.

**Figure 3:**
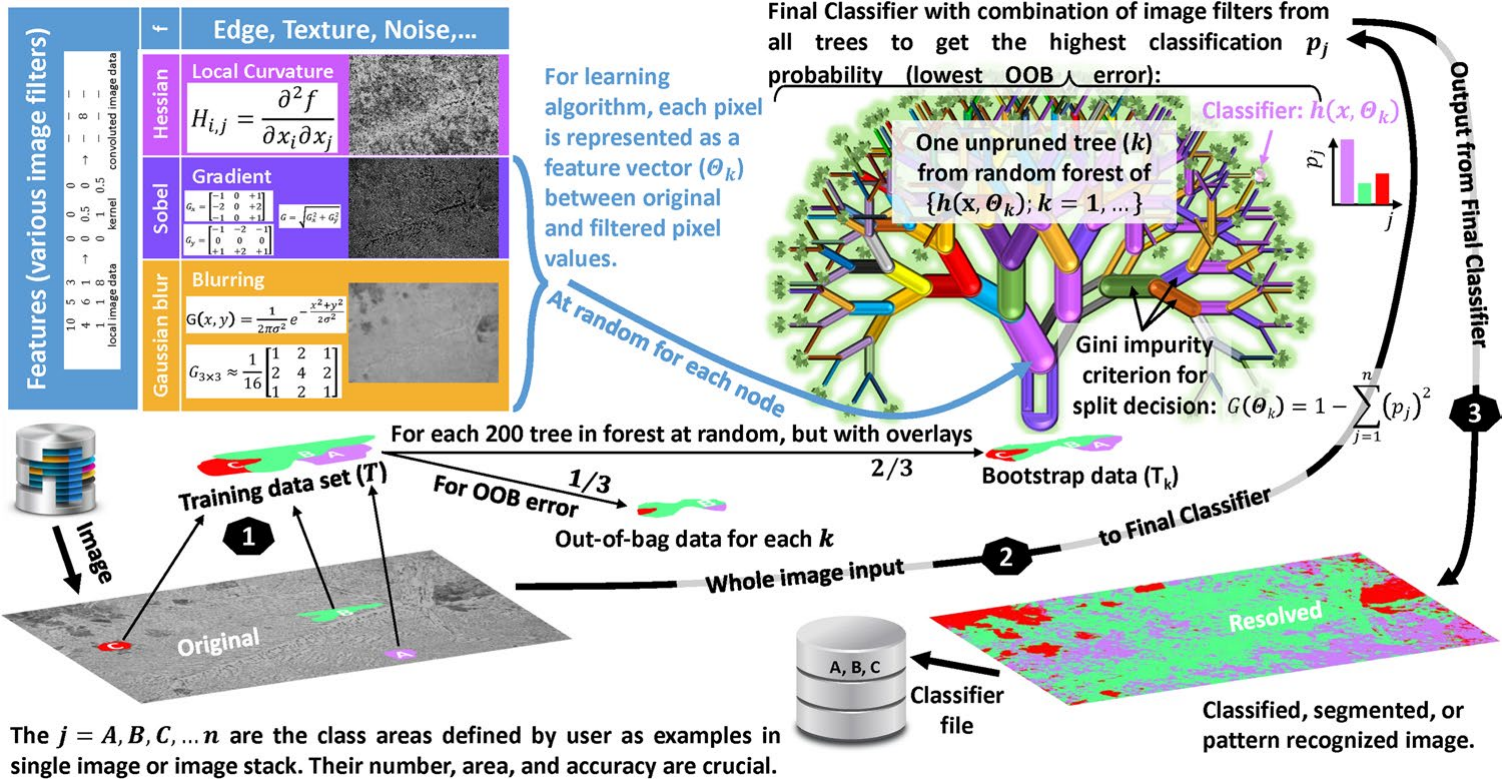
Scheme of image segmentation with machine learning Random Forest statistical algorithm (see text for more details).

The basic outline of the method is shown in Fig. 3. It belongs to an ensemble-type supervised learning. As such, the examples of image pixels (areas) for different j (microstructures) are given by user to form the training data set (T) from single image or image stack. The bootstrap data (*T*_*k*_), which are used to construct each decision tree (k), are chosen at random from T, but with overlays. In each bootstrapping process, approximately one-third of the T are left unused, which forms the out-of-bag (OOB) dataset for later utilization in estimation of the prediction error of machine-learning segmentation.

The individual k is grown by applying the randomly selected feature from image filter/kernel set (*N*_*f*_) to the *T*_*k*_, i.e. by transforming the image input vectors (**x**) to feature vectors (Θ_*k*_). After such filtering, at every split of the node (branches), the threshold-based yes/no split function is used to assign each pixel to particular j. The probabilities (*p*_*j*_) of correct classification are calculated for each j by dividing the numbers of assigned and actual pixels in *T*_*k*_. From calculated *p*_*j*_ for parent and child nodes, the Gini impurity criteria [${\rm{G}}({{\rm{\Theta }}}_{k})=1-{\sum }_{j=A}^{n}{({p}_{j})}^{2}$] are estimated. The *k* is allowed to grow only if child node has lower G(Θ_*k*_) value for some j. In other words, only the selected image filters and optimized split function parameters at each node, that lead to information gain (better classification), are selected for the k to grow until the predefined threshold for G(Θ_*k*_) or number of branches is reached.

After all k are grown, the leaf nodes [*L*(*l*_1_, …, *l*_*n*_)] contain the probability distributions *p*(*j*|*L*) of pixels for each j. These statistics will be used during the decision-making process at classification stage. The k-Classifier [*h*(**x**, *Θ*_*k*_)] stores the information on Θ_*k*_ for each node, the optimised binary test for each split, and *p*(*j*|*L*_*k*_). The collection of such *h*(**x**, *Θ*_*k*_) from all *k* constitutes the Random Forest Classifier [{*h*(**x**, *Θ*_*k*_); *k* = 1, …}]. To check its quality, the OOB data are used and the OOB error is calculated by [${\sum }_{k}I(h({\bf{x}},{\Theta }_{k})=j;\,(y,{\bf{x}})\notin {T}_{k})/{\sum }_{k}I((y,{\bf{x}})\notin {T}_{k})$] with indicator function [*I*(·)], i.e. by estimation of the proportion of times averaged over all cases that estimated j is not equal to the true class (*y*) in the OOB dataset.

At classification stage, the pixel that arrives to *L*_*k*_ after all filtering and binary tests stages has *p*(*j*|*L*_*k*_) for each *k*. To make a decision on j assignment for that pixel, each *k* casts a unit vote for the most popular class. These votes from all *k* cast a unit vote too. As a result, that pixel is finally classified to the particular j. If OOB error and the result of the image segmentation for the training data are satisfactory to the user, then such {*h*(**x**, *Θ*_*k*_); *k* = 1, …} can be applied to classify/segment the images of actual interest for statistical analysis. If not, the Classifier training process can be repeated by adding/revising of training data, image filters (edge, texture, noise, et. cet.), and tree/forest scales. Actually, each *k* is just a weak Classifier, but the forest of {*h*(**x**, *Θ*_*k*_); *k* = 1, …} can be strong and unbiased thanks to an ensemble effect together with randomness in bootstrapping (de-correlating trees) and filter selections.

Though the expertise in steel microstructures is needed at the Classifier training step, but after that, even novice users can apply the well-trained Classifier for automatic segmentation of their image or image stack. This opens the possibilities to work with large image data volumes for materials informatics and materials integration goals across the fields. Such technique should also find the widespread use in industrial quality control and in research laboratories.

For appropriate image analysis of microstructures and from a viewpoint of practical industrial applicability, all samples in present work were prepared by a conventional polishing and subsequent etching^34^ and observed by using an optical microscopy.

## Results and Discussion

### Segmentation with machine learning

**Figure 4:**
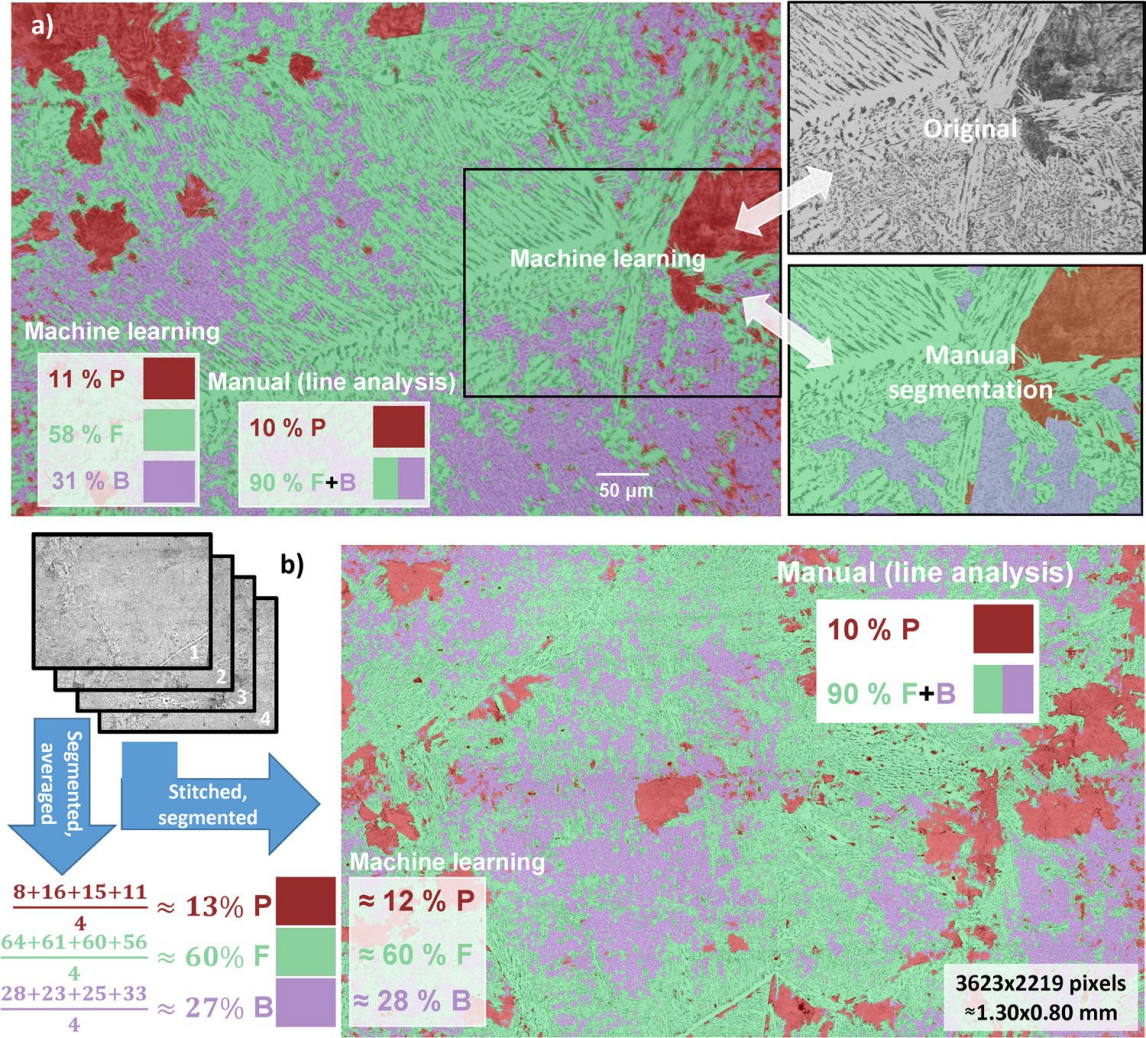
Examples of machine learning with Random Forest algorithm for automated pattern recognition in optical microscopy images of metallurgical samples. (**a**) The automated segmentation on single image is on the left. The small part of it and manual segmentation on this part are depicted in the right for comparison. (**b**) The application of the Random Forest Classifier on image stack or stitched image for accurate estimation of microstructure area/volume percentage from large imaging area.

Figure 4(a) shows the example of image segmentation with Random Forest algorithm on F = F_sp_ + F_all_, B, and P microstructures for steel A from Fig. 2(b), but for different and larger area. The original image on the background is overlaid with segmented colour-coded areas for F, B, and P. The calculated phase percentages on inserts of Fig. 4(a) are also given for comparison with manual linear analysis of P and F + B phases. The good agreement will be further validated below by using a much larger imaging area.

In addition, the part of the original image from Fig. 4(a) and its manual segmentation results are shown for comparison. Here, it should be stressed that manual area segmentation is not practical in real-world applications due to its significant labour and time demands. Nevertheless, it was confirmed that machine learning and expert-level manual segmentations are in excellent agreement with only minor inconsistencies in detecting of small microstructure areas.

Figure 4(b) shows the segmentation, which was performed on 3623 × 2219 pixel image (~1 mm^2^) by stitching^35^ and slight cropping of four 1920 × 1440 pixel images with ~0.36 μm/pixel scale. The Classifier was created on one of them and applied to others and to the stitched version. As demonstrated in Fig. 4(b), the accuracy of microstructure percentage estimation become comparable with manual linear analysis by averaging of segmentation results for image stack (see percentage deviations within the stack) or by using of stitched image with large imaging area. Both methods produce practically identical results. This demonstrates the good reproducibility and necessity to image the appropriate area size for accurate microstructure percentage estimation.

Table 1 summarises the comparison between manual (linear) and automated (Random Forest segmentation and empirical calculation) image analyses for several A-steels obtained at different cooling rates. Note that additional accurate segmentation on F and B areas was also achieved with machine learning compared to the manual line analysis (see Fig. 4 and Table 1). For the variety of the steel microstructures such as P + F, P + F + B, and B + M, the present automated segmentations show a very good agreement with manual analysis, which proofs the practical aspect of used segmentation method for various steels (see listed errors, i.e. the cross-validation by manual linear analysis).

**Table 1:** Experimental conditions, mechanical properties and volume percentages of microstructure phases for several A-steels (see chemical composition in Fig. 2) measured by manual and automated image analysis.

CR K/s	Hv	V_manual_ %					V_auto_ %					Area mm^2^
		P	F	B	F + B	M	P	F	B	F + B	M	
0.3		25	75		100		21(4)	79(4)		100		5.77
1		24	76		100		22(2)	78(2)		100		1.12
3		10	**	**	90		10(0)	58	32	90(0)		1.34
10	313			45^*^					43(2)		57	0.50
15	334			35^*^					31(4)		69	0.50

The CR, Hv, V_manual_, V_auto_, and Area are the cooling rate, Vickers hardness, volume percentage of phases by manual line analysis, volume percentage of phases by automated Random Forest segmentation, and analyzed image area for V_auto_ estimation, respectively. The numbers in brackets are the errors between manual and automated analyses.

*Calculated from empirical equation of B and M hardness^43^.

**F and B were not separately counted.

In Fig. 4 the OOB error was ~1% with more than 70 features and class homogenization (balanced distribution of j pixels) used^30^. Due to the nature of the Random Forest, the larger *N*_*f*_ produces the lower OOB error^28^. For example, without class homogenization, the Classifier created by using original image in Fig. 4(a) with only Gaussian Blur, Hessian, Sobel, Difference of Gaussians, and Membrane Projection filters gave the OOB error of ~6%. By using all *N*_*f*_, the OOB error decreased to ~2%. In above comparison, the same T (P = 16346, F = 86957, and B = 3850 pixels), k = 200, two random features per node, two decimal places for calculated precision, and filter parameter (standard deviation, etc.) ranges were used. However, the larger *N*_*f*_ increased the computation time for Classifier creation by ~5 times. Among used *N*_*f*_, the Anisotropic Diffusion, Gabor, Kuwahara, and Bilateral filters were mostly accounted for the time difference. The runtime and OOB error also scale with k^33,36^, but later one typically saturates for k > 100. At classification stage, with present ~250 MB Classifier file size, it takes ~4 min and ~10 GB additional RAM to segment the 2352 × 1568-pixel image on two-CPU Optiron 6128 workstation.

For industrial applications, when the computation time is important, the Classifier could be fine-tuned with particular T, *N*_*f*_, filter parameters, k, and OOB error, which are suitable for the task of acceptable segmentation quality. In principle, the cross-validation is performed internally in Random Forest algorithm during the run by using the OOB dataset and lower OOB error is a good indicator of better Classifier quality. However, the main challenge is at Classifier application stage on datasets, which may differ strongly in image quality. In this regard, the development/use of appropriate cross-validation protocols by automated or human inspection with sampling analysis may still be needed. The major limitation for practical industrial applications is in supervised-learning nature of the Random Forest algorithm. Therefore, the best way is, first, to analyse the image data in terms of j types with some automated unsupervised-learning algorithm or statistical image analysis, and then to choose the appropriate Random Forest Classifier for this j combination from corresponding library. These are the interesting and important topics for future research and development.

Apart from general image quality and colour scale, the Classifier is sensitive to the image spatial scale, contrast, and j present. For example, it will be wrong to apply the Classifier for sample in Fig. 4(a) on Fig. 2(a) due to different μm/pixel scale and absence of B microstructure. From practical view point, the high numerical aperture (NA) microscope objective (NA = 0.8–0.9) could be used to collect the image dataset over ~1 mm^2^ sample area. Typically, this will allow to create one Classifier for all phases present in particular sample zone of interest for statistical analysis of metallurgical material. As for the contrast variations, the image histograms should not differ much during picture collections and images should be run through the standardized brightness/contrast adjustments afterwards^37^. Nowadays, the automatic illumination/exposure control for consistently good image quality is a common feature with industrial optical microscopes. Nevertheless, more robust split functions could be also used or developed in this regard^38^.

### Segmentation with Euclidean distance conversion

**Figure 5:**
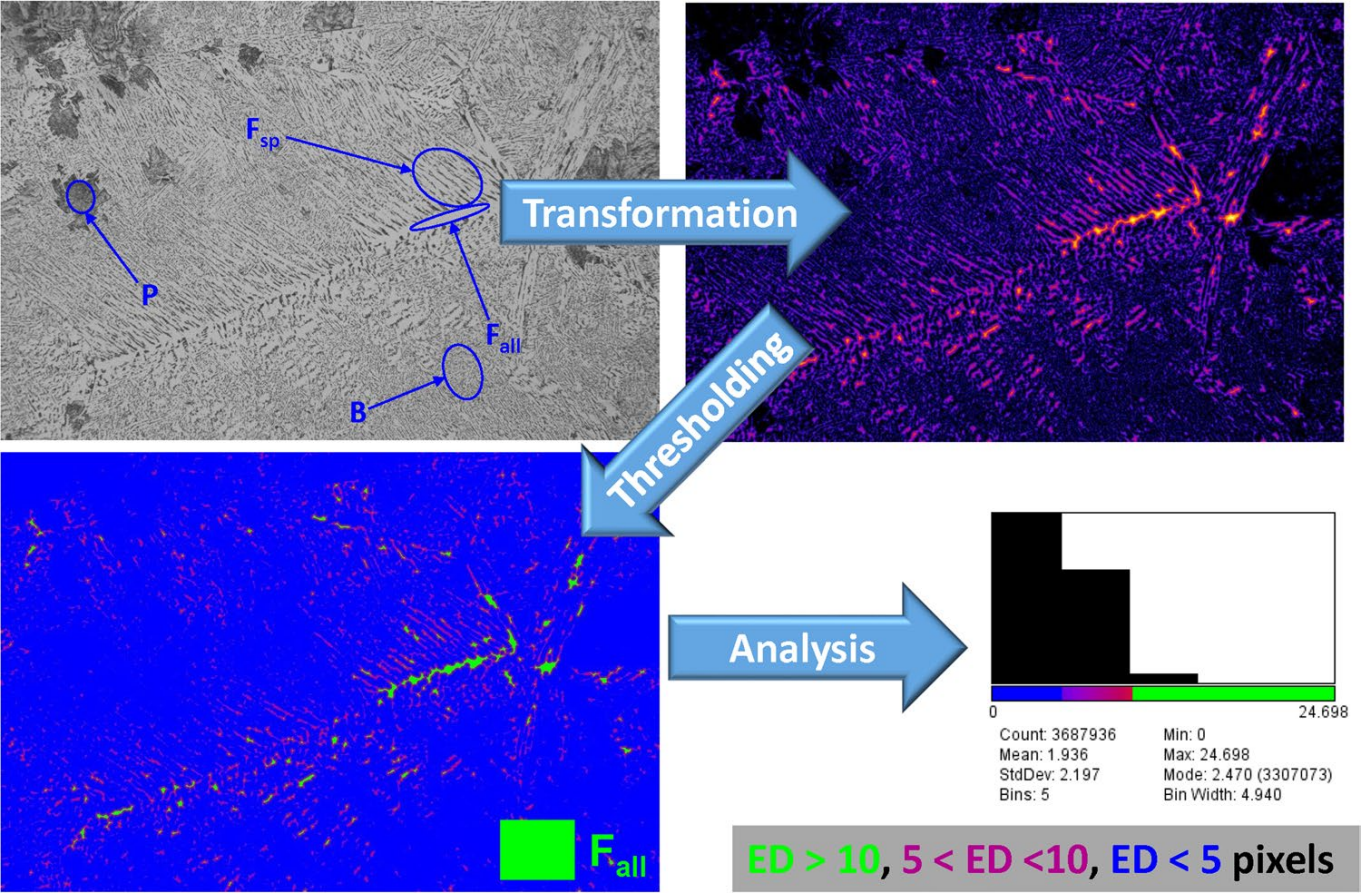
The image analysis protocol with Euclidean distance conversion technique for ferrite sub-phase segmentation in steels.

To extract more information from Fig. 4(a), additional image processing and analysis techniques developed for different scientific fields could be also applied here. For example, the scheme of protocol with Euclidean distance (ED) conversion is shown in Fig. 5 for highlighting/segmenting of F_all_. In this method, each foreground pixel (grey one) in original image is replaced with a colour value equal to that pixel’s distance from the nearest background pixel (black one)^39,40^.

Because of higher nucleation temperature of F_all_ compared to F_sp_ and slow cooling rate^1^, the diffusion distance of carbon from F_all_ to A becomes larger than the width of F_sp_ and it precipitates as cementite (black pixels) between F_sp_. As a result, the F_all_ areas appears brighter in ED map (see Fig. 5), i.e. the F_all_ corresponds to wider areas between black pixels. Then, depending on user’s criteria/thresholding on estimated ED map, the coloured areas could be assigned to the different types of F and studied quantitatively, for example, by additional segmentation of F_all_ from F_sp_.

This analysis is important since plastic deformation of the soft F_all_ can relax the local stress concentration and could affect the crack initiation/propagation in steels^41^. As it is seen from Fig. 4(a), there are no clear boundaries between F_all_, F_sp_, and B. Therefore, the manual segmentation could be a challenge even for an expert. In this regard, the further correlations between mechanical properties and ED analysis results could help to find the mathematical criteria for correct segmentation of some phases.

### Segmentation with structure tensor extraction

**Figure 6:**
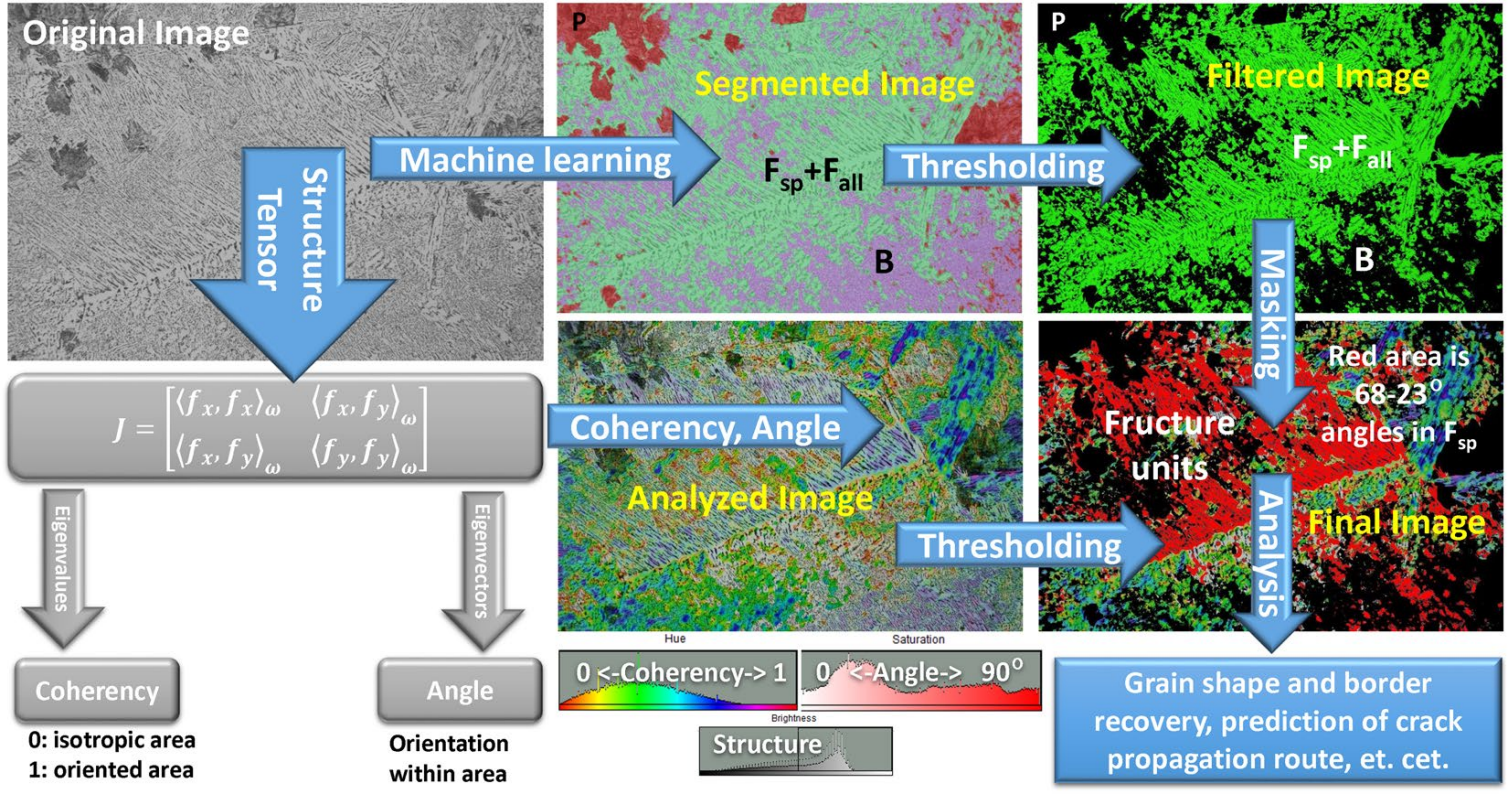
The image analysis protocol with structure tensor estimation for spatial anisotropy segmentation of fracture units in steel grains.

Figure 6 shows another example of protocol scheme for detecting the spatial anisotropy in images of steels. It utilizes the evaluation of the structure tensor (J) in a local neighbourhood, which is commonly used in the field of image processing and is further developed for biological applications^42^. In this method, the continuous spatial derivatives of cubic B-spline interpolation along X and Y axes for the input image are used in evaluations of coherency and orientation maps from J eigenvalues and eigenvectors, respectively.

For metallurgical samples, this analysis could highlight/segment the areas with isotropic (B and P) and anisotropic (F_sp_) microstructure patters (depending on image scale and J Gaussian window). In combination with machine learning results of image segmentation in Fig. 4(a), the B and P areas are first masked out and then the dimensions of microfracture unit, which is responsible for cleavage fracture (F_all_ area oriented in same direction on Final Image in Fig. 6), could be highlighted. This was done by filtering of the microstructure orientation angles at 60° from grain boundaries. This angle corresponds to the cementite and dislocation growths during F_sp_ formation.

Since steel cracking is affected by grain geometry and rolled steels display spatial anisotropy of grain alignments, such fracture unit and grain visualizations could be used to correlate the macroscopic mechanical and microscopic structural properties in more details in order to predict the crack propagation routes.

## Concluding Remarks

To our best knowledge, we are reporting for the first time the highly accurate, practical, and fast image segmentation with machine learning for microscopy images of metallurgical samples. This technique in combination with appropriate image processing/segmentation protocols, including image filtering, thresholding, and other mathematical/statistical methods, could revolutionize the image analysis in metallurgy field by creating the classifier/protocol libraries/databases for typical microstructure patterns. Table 2 summarizes the problems, methods, and applicability. In summary, we can conclude that automated expert-level analysis is getting to be quite feasible by using an appropriate method.

**Table 2:** Summary of the image segmentation methods for metal materials paradigm.

Problems	Tools	Comments/Examples
Segmentation of metal microstructures	Expert-supervised Machine Learning by using Random Forest classification with a suitable set of image filters	Most versatile method to segment F, P, B, M, M-A, et. cet. of steel microstructures from image data (see Fig. 4). The segmentation quality is reasonably practical to obtain the statistics on the volume fraction of each phase.
Highlighting of particular microstructure	Euclidean distance conversion	Powerful image processing tool to segment, visualize, and analyze the different F sub-phases (see Fig. 5), which is a challenge even for an expert-level machine learning.
Spatial orientation/anisotropy of microstructures	Structure tensor extraction	Useful image processing tool to visualize, segment, and analyze the steel microstructures with anisotropic orientations of cementite or dislocations in F_sp_, B, P, and M microstructures as well as of rolled steel grains (see Fig. 6).
Quantitative characterization of microstructures from large image data volumes	Combination of above tools with thresholding and various filtering techniques	It is important to create the classifier file library and protocols with above tools for the fast, reliable, and routine segmentations by common user (ongoing work, see Acknowledgments).

## References

[1] Bhadeshia, H. K. D. H. & Honeycombe, R. *Steels. Microstructure and Properties* (Elsevier, 2006).

[2] Krauss, G. *Steels: Processing, Structure, and Performance* (ASM International, 2005).

[3] Bramfitt, B. L. *Structure/Property Relationships in Irons and Steels, Metals Handbook Desk Edition* (ed. Davis, J. R.) 153–173 (ASM International, Second Edition, 1998).

[4] Kumar A, Singh SB, Ray KK (2008). Influence of bainite/martensite-content on the tensile properties of low carbon dual-phase steels. Mater. Sci. & Eng. A.

[5] Zare A, Ekrami A (2011). Influence of martensite volume fraction on tensile properties of triple phase ferrite–bainite–martensite steels. Mater. Sci. & Eng. A.

[6] Witten, I. H. & Frank, E. *Data mining: practical machine learning tools and techniques* (Morgan Kaufmann series in data management systems, Second Edition, 2005).

[7] Schneider CA, Rasband WS, Eliceiri KW (2012). NIH Image to ImageJ: 25 years of image analysis. Nature Methods.

[8] Collins TJ (2007). ImageJ for microscopy. BioTechniques.

[9] Schindelin J (2012). Fiji: an open-source platform for biological-image analysis. Nature methods.

[10] Ma Z, Tavares JMRS, Jorge RN, Mascarenhas T (2010). A review of algorithms for medical image segmentation and their applications to the female pelvic cavity. Comput. Methods Biomech. Biomed. Engin..

[11] Oliveira RB (2016). Computational methods for the image segmentation of pigmented skin lesions: a review. Comput. Methods Programs Biomed..

[12] Jodas DS, Pereira AS, Tavares JMRS (2016). A review of computational methods applied for identification and quantification of atherosclerotic plaques in images. Expert Syst. Appl..

[13] Burikova K, Rosenberg G (2009). Quantification of Microstructural Parameter Ferritic-Martensite Dual Phase Steel by Image Analysis. Metal 2009, Hradec nad Moravici, May.

[14] Dengiz O, Smith AE, Nettleship I (2005). Grain boundary detection in microstructure images using computational intelligence. Comput. Ind..

[15] Chmiela J, Słota D, Szala J (2009). Multiscale description of the inhomogeneity of multiphase materials. Mater. Charact..

[16] Kim D, Liu JJ, Han C (2011). Determination of steel quality based on discriminating textural feature selection. Chem. Eng. Sci..

[17] Dutta S (2014). Characterization of micrographs and fractographs of Cu-strengthened HSLA steel using image texture analysis. Measurement.

[18] Komenda J (2001). Automatic recognition of complex microstructures using the Image Classifier. Mater. Charact..

[19] DeCost BL, Holm EA (2015). A computer vision approach for automated analysis and classification of microstructural image data. Comput. Mater. Sci..

[20] Adachi Y, Taguchi M, Hirokawa S (2016). Microstructure Recognition by Deep Learning. Tetsu-to-Hagané.

[21] Taguchi M, Hirokawa S, Yasuda I, Tokuda K, Adachi Y (2017). Microstructure Detection by Advanced Image Processing. Tetsu-to-Hagané.

[22] De Albuquerque VHC, Cortez PC, Alexandria AR, Tavares JMRS (2008). A new solution for automatic microstructures analysis from images based on a backpropagation artificial neural network. NDT & E Int..

[23] De Albuquerque VHC, Alexandria AR, Cortez PC, Tavares JMRS (2009). Evaluation of multilayer perceptron and self-organizing map neural network topologies applied on microstructure segmentation from metallographic images. NDT & E Int..

[24] De Albuquerque V, Tavares JMRS, Cortez P (2010). Quantification of the microstructures of hypoeutectic white cast iron using mathematical morphology and an artificial neural network. IJMMP.

[25] De Albuquerque VHC, Reboucas Filho PP, Cavalçante TS, Tavares JMRS (2010). New computational solution to quantify synthetic material porosity from optical microscopic images. J. Microsc..

[26] Albuquerque VHCD, Silva CC, Menezes TIDS, Farias JP, Tavares JMRS (2011). Automatic evaluation of nickel alloy secondary phases from SEM images. Microsc. Res. Tech..

[27] Papa JP, Nakamura RYM, de Albuquerque VHC, Falcao AX, Tavares JMRS (2013). Computer techniques towards the automatic characterization of graphite particles in metallographic images of industrial materials. Expert Syst. Appl..

[28] Breiman L (2001). Random forests. Machine Learning.

[29] Breiman L (1996). Technical Note: Some Properties of Splitting Criteria. Machine Learning.

[30] Arganda-Carreras, I., *et al*. Trainable Weka Segmentation: a machine learning tool for microscopy pixel classification. *Bioinformatics* (Oxford, England) (2017). 10.1093/bioinformatics/btx180 28369169

[31] Vyas N, Sammons RL, Addison O, Dehghani H, Walmsley AD (2016). A quantitative method to measure biofilm removal efficiency from complex biomaterial surfaces using SEM and image analysis. Scientific Reports.

[32] Staniewicz L, Midgley PA (2015). Machine learning as a tool for classifying electron tomographic reconstructions. Adv. Struct. Chem. Imaging..

[33] Ko BC, Kim SH, Nam J-Y (2011). X-ray image classification using Random Forests with local wavelet-based CS-local binary patterns. J. Digit. Imaging.

[34] Ikawa H, Oshige H, Tanoue T (1980). Study on the martensite-austenite constituent in weld-heat affected zone of high strength steel. J. Jap. Weld. Soc..

[35] Preibisch S, Saalfeld S, Tomancak P (2009). Globally optimal stitching of tiled 3D microscopic image acquisitions. Bioinformatics.

[36] Wright MN, Ziegler A (2017). A fast implementation of Random Forests for high dimensional data in C++ and R. J. Stat. Softw..

[37] Ferreira, T. & Rasband, W. ImageJ user guide IJ 1.46r. http://imagej.nih.gov/ij/docs/guide (2012).

[38] Gall, J., Razavi, N. & Van Gool, L. *An Introduction to Random Forests for Multi-class Object Detection* (eds Dellaert, F., Frahm, J. M., Pollefeys, M., Leal-Taixé, L. & Rosenhahn, B.) *Outdoor and Large-Scale Real-World Scene Analysis. Lecture Notes in Computer Science* **7474**, 243–263 (Springer, Berlin, Heidelberg 2012).

[39] Dougherty, B., Schindelin, J., Doube, M., Domander, R. & Hiner, M. Fiji: Local Thickness. https://imagej.net/Local_Thickness (2017).

[40] Saito T, Toriwaki J (1994). New algorithms for Euclidean distance transformation on an n-dimensional digitized picture with applications. Pattern Recognition.

[41] Xua P, Bai B, Yin F, Fang H, Nagai K (2004). Microstructure control and wear resistance of grain boundary allotriomorphic ferrite/granular bainite duplex steel. Mater. Sci. & Eng. A.

[42] Rezakhaniha R (2012). Experimental investigation of collagen waviness and orientation in the arterial adventitia using confocal laser scanning microscopy. Biomech. Model Mechanobiol..

[43] Yurioka N, Okumura M, Kasuya T, Cotton HJU (1987). Prediction of HAZ hardness of transformable steels. Metal Construction.

